# THE COMPARISON BETWEEN THE EFFICACY OF HIGH DOSE ACYCLOVIR AND ERYTHROMYCIN ON THE PERIOD AND SIGNS OF PITIRIASIS ROSEA

**DOI:** 10.4103/0019-5154.70672

**Published:** 2010

**Authors:** Amirhooshang Ehsani, Nafiseh Esmaily, Pedram Noormohammadpour, Siavash Toosi, Alireza Hosseinpour, Mahbobeh Hosseini, Shima Sayanjali

**Affiliations:** *From the Department of Dermatology, Tehran University of Medical Sciences, Razi Hospital Iran*; 1 *From the Department of Internal Medicine, Baquitallah University of Medical Sciences, Baquitallah Hospital, Tehran, Iran*

**Keywords:** *Acyclovir*, *erythromycin*, *pitiriasis rosea*

## Abstract

**Background:**

Pityriasis Rosea (PR) is an acute inflammatory and self-limiting skin disorder, sometimes with troublesome symptoms. To date, there are few treatments available for this disorder.

**Aim:**

Compare the traditional treatment with erythromycin to a newly introduced antiviral treatment acyclovir for PR.

**Materials and Methods:**

Patients with clinically confirmed diagnosis of PR, matching our exclusion criteria, were enrolled. They were randomized in two groups that received high-dose oral acyclovir or erythromycin. The participants were evaluated two, four, and eight weeks after commencement of the study and followed for one year.

**Results:**

A total of 30 patients including 15 males and 15 females completed the study. After eight weeks, 13 patients in the acyclovir group experienced complete response, while in the erythromycin group only six patients had complete response (*P* < 0.05). Also, patients in the acyclovir group experienced faster resolution of pruritus in comparison with the erythromycin group (not significant). No adverse drug reaction was detected in both groups.

**Conclusion:**

It seemed that a high-dose of oral acyclovir was a safe and effective therapy for PR, although this remained to be confirmed in larger studies.

## Background

Pityriasis rosea (PR) is an acute inflammatory and self- limiting disease of the skin. It typically begins as a single thin oval scaly plaque called a herald patch, and is typically asymptomatic.[1] PR is common among patients visiting dermatologists. The incidence of PR varies from 0.39[2] to 4.80[3] per 100 dermatological patients. The prevalence of PR is estimated at approximately 0.6% between 10 and 29-year-old people,[4] with an Male/Female ratio of about 1/1.43.[5] There are a number of studies in favor of an infectious etiology for this disorder,[6,7] and now it is believed that PR is induced by some of the Human Herpes Virus members, mainly HHV7 and HHV6.[8–10] Oral erythromycin is reported to be of benefit to patients with PR,[11] although recent clinical experiences suggest that the use of macrolids including erythromycin and azithromycin are of no value in the treatment of PR.[12–14] Recent studies are focused on using antiviral agents in this disorder, mainly acyclovir.[15] According to this study, it seems that acyclovir is an effective and safe therapy for treating PR.

We were unable to find a study comparing these two drugs. The aim of this study is to compare the efficacy of the traditional treatment with erythromycin and the recently introduced treatment with acyclovir.

## Materials and Methods

This randomized clinical trial was conducted for a period of one year from May 2007 to April 2008. Patients with diagnosis of PR, confirmed clinically by two academic dermatologists, were selected for the study, if they visited our clinic during the first week of their disease and if they had extensive generalized lesions. An informed consent was taken from each patient or parent for inclusion in the study. Patients were excluded if they were pregnant or breast feeding women, or had any history of sensitivity to acyclovir or erythromycin, and renal or hepatic impairment, or were suspected to have fungal infection, psoriasis, or eczema. *Venereal Disease Research Laboratory* (VDRL) test was performed on all patients and positive results were confirmed by the fluorescent treponemal antibody absorbed (FTA-ABS) test, to exclude second-stage syphilis. The patients were randomized in two groups, A and B. Group A received high-dose acyclovir (4 gr daily, in five divided doses, for 10 days) while group B received oral erythromycin (400 mg QID for 10 days). Acyclovir and erythromycin used in the study were in similar white pill forms, with the same packaging. The patients were visited two, four, and eight weeks after commencement of the study, to evaluate response to treatment and then followed for one year to detect any recurrences. They were evaluated for improvement in symptoms (mainly pruritus), appearance of new lesions or resolution of previous ones, and increase or decrease in erythema and scaling. Count of lesions on the body was documented on every visit, for further evaluation of response to treatment. The responses were categorized as:

- Complete response: No new lesions followed by disappearing of all previous lesions, with or without residual post-lesional pigmentation
- Partial response: Few new lesions plus regression or disappearing of some previous lesions
- No response: No regression of lesions, along with appearance of new lesions.

### Statistical analysis

Student t-test was used for comparing means, while the chi-square test was used for comparing differences in proportions wherever necessary. *P* < 0.05 was considered significant.

## Results

The participants in the study were 30 patients with PR, who passed our inclusion and exclusion criteria. The average age of the patients who attended this study was 32.9 ± 16 years; 15 (50%) female and 15 (50%) male.

### Characteristics of the two groups

Table 1 shows the demographic characteristics of the two groups. As shown in the table, there were no significant differences between them.

**Table 1:** Demographic characteristics of patients

Parameters	Acyclovir group (n = 15, 50%)	Erythromycin group (n = 15, 50%)	*P* value
Age:			
< 25 years	3	4	NS
25 – 35 years	5	6		
> 35 years	7	5	
Sex:			
Male	8	7	NS
Female	7	8
Summary	15	15	

NS: Not statistically significant

Six patients in the erythromycin group and eight patients in acyclovir group had pruritus before the commencement of the study.

### Response to drugs

At the end of the study, in group A, which received high- dose acyclovir, 13 patients had complete response, while in group B, treated with erythromycin, only six patients had complete response (P < 0.05). Table 2 shows the rate of response to the prescribed drugs in the two groups.

**Table 2:** Response to prescribed drugs

	First visit (2 weeks after treatment)		Second visit (4 weeks after treatment)		Third visit (8 weeks after treatment)	
	Acyclovir	Erythromycin	Acyclovir	Erythromycin	Acyclovir	Erythromycin
Complete response	0	0	8	1	13*	6
Partial response	14	9	7	12	2	9
No response	1	6	0	2	0	0
Pruritus	0	6	0	2	0	1
Summary	15	15	15	15	15	15

*: Statistically significant

After eight weeks, in the patients treated with acyclovir, two had partial response, while in the erythromycin group, nine had partial response (P > 0.05). Pruritis is one of the most distressing symptoms of PR. As we can see, in group A, pruritus in all patients resolved after two weeks, while in group B two patients after four weeks and one patient after eight weeks had pruritus, although this was not significant.

## Discussion

In our study, the male to female ratio is about one in both treatment groups. None of our patients in both groups achieved complete response during the first two weeks of treatment. This is contrary to some other trials[11,15,16] that reported complete response after two weeks of treatment in their subjects. After eight weeks, the rate of complete response in patients who received high-dose acyclovir was significantly higher than their matches who received oral erythromycin. This is in agreement with the studies that reported the efficacy of acyclovir in treating PR[15] and questioned the effect of erythromycin in this disorder.[12,13] Nowadays, there are increasing evidences toward confirming the role of the human herpes virus.[17–19] There is an important note to consider with regard to the effect of acyclovir on different herpes virus family members. To date, it has been seen that only high doses of acyclovir have had a suppressing effect on HHV-6[20–22] and the same is correct about HHV-7. In fact HHV-7 is less sensitive to acyclovir than HHV-6.[23,24] These studies have shown that antiviral drugs such as Cidofovir and Foscarnet[25–27] may be more effective against HHV-6, 7. Considering the high rate of side-effects of these drugs and according to the selflimiting nature of PR, in many cases we preferred to use high-dose acyclovir as a safer alternative, to evaluate it’s effects on relieving the symptoms of PR. A significantly higher response to high-dose acyclovir in our study may be another clue to confirm the positive role of this drug in eliminating some manifestations of PR. According to a small number of patients included in our study and the clinical confirmation of PR diagnosis, we suggest further trials with a larger number of participants, and possibly a pathologically confirmed diagnosis of PR, to confirm our results. Acyclovir is probably a safe treatment in patients with PR according to their age range, but its efficacy must be confirmed in larger studies with higher sample sizes.
